# Assessment of Coolant Delivery Techniques for Irrigation During Bone Drilling: A Cadaveric Observation

**DOI:** 10.5704/MOJ.2011.037

**Published:** 2020-11

**Authors:** ML Abdul-Rashid, HL Tan, D Pancharatnam

**Affiliations:** Department of Orthopaedic Surgery, University of Malaya, Kuala Lumpur, Malaysia

Dear Editor,

We would like to share an unexpected finding related to a common orthopaedic procedure. Erickson *et al* reported that temperature above 47oC for one minute is the critical threshold for bone necrosis^1^. Modification of drill bits designs (cutting surface, flutes/helix angle), drilling techniques (drilling speed, drilling time, pre-drilling) and use of coolant has been recommended to lower this risk^2^. Although many orthopaedic and trauma surgeons are faithfully irrigating the drill bit / drill sleeve with saline during bone drilling for this purpose, we do not even know whether the coolant ever reach the bone / drill bit interface, especially during minimally invasive percutaneous procedures. Benington *et al* and Sener *et al* showed that external saline irrigation was effective to prevent bone necrosis for dental procedures, but there was no similar study on clinical orthopaedic models^3,4^.

**Fig. 1:**
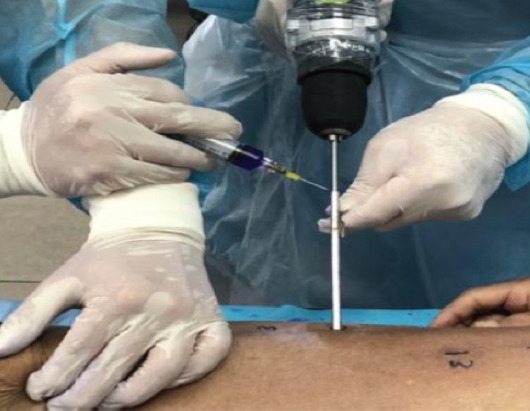
Delivering of coolant on drill bit through drill sleeve.

**Fig. 2:**
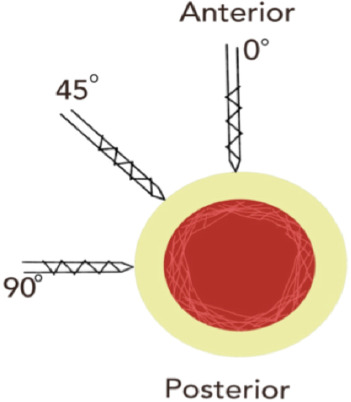
Angular variation of drilling based on the axial plane of femur and tibia.

We decided to investigate whether saline sprayed over the incision wounds or outer end of drill sleeves wound reach the interface between bone and drill bit during bone drilling (Fig. 1). We chose different sites corresponding to safe zones of external fixator for the femur and tibia, and introduce a new 3.5mm drill bit / corresponding drill sleeve at angles of 0°, 45° and 90° to the vertical line (Fig. 2) through a 1cm incision wound. Drilling were performed for about 5 seconds, irrigated with 10ml of saline (with blue dye). The drilling path were then dissected down to the bone for examination.

**Fig. 3:**
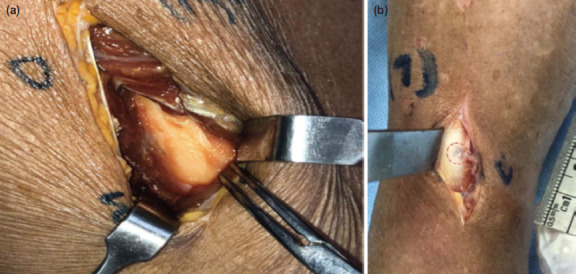
Exposure of wounds over mid-shaft femur and distal tibia. (a) Dye only detected over superficial layers of soft tissue of mid-shaft. (b) Dye detected at bone / drill bit interface at distal tibia: Irrigation through (in dotted circle).

We noted that there was no trace of blue dye on the bone surface for the whole femur and proximal end of the tibia (Fig. 3a). Blue dye can be seen on bone surface on the medial surface of shaft and distal tibia, corresponding to 0° and 45° angulation of the drill bit / sleeve (Fig. 3b). This observation showed that we should not rely on saline irrigation to reduce the bone temperature during bone drilling, unless the bone is exposed (open reduction internal fixation) or when the soft tissue is very thin. Interestingly our soft tissue assessment showed a trend that the saline penetrated deeper at lower angle (more vertical) of drilling and less with bigger angles (more horizontal).
